# Is Aging a Failure to Resolve? Integrating Damage Responses, Regenerative Decline, and Geroscience Interventions

**DOI:** 10.3390/ijms27208987

**Published:** 2026-10-10

**Authors:** Piotr Paweł Chmielewski, Bartłomiej Strzelec

**Affiliations:** 1 Division of Anatomy, Department of Human Morphology and Embryology, Faculty of Medicine, Wroclaw Medical University, 6a Chalubinskiego Street, 50-368 Wrocław, Poland; 2 University Center of General and Oncological Surgery, Wroclaw Medical University, 50-556 Wroclaw, Poland; bartlomiej.strzelec@umw.edu.pl

**Keywords:** aging, biological age, gerontology, geroscience, tissue damage response, wound healing, inflammaging, cellular senescence, cGAS-STING, regenerative medicine

## Abstract

Biological aging is accompanied by persistent tissue-damage signaling, chronic inflammation, extracellular matrix remodeling, cellular senescence, and declining regenerative capacity. Taken together, these features resemble an injury response that is repeatedly initiated but fails to progress reliably through clearance, reconstruction, and functional recovery. This resemblance motivated the proposal that aging is a wound that never starts healing. Here, we develop that proposal into a testable failed-resolution framework in which some age-related dysfunction reflects damage responses that exceed organ-specific capacities for intracellular quality control, efferocytosis, immune termination, matrix normalization, and cell replacement. Its central predictions are that (i) impaired recovery kinetics will forecast functional decline beyond established measures of biological age, (ii) restoring resolution will improve tissue function beyond suppressing inflammatory markers, and (iii) efficacy will depend on treatment phase and tissue-specific failure mode. We specify which age-related processes lie outside the framework and propose measurements spanning damage burden, danger sensing, resolution competence, regenerative reserve, organ function, and recovery after standardized challenge. Supporting evidence is predominantly preclinical, whereas human studies provide mechanistic findings, target engagement, or domain-specific effects. Failed resolution thus offers a falsifiable research program for testing tissue recovery as a therapeutic target in aging.

## 1. Introduction

Aged mammalian tissues display features characteristic of early injury responses, providing a biological basis for the hypothesis that aging resembles a wound that never starts healing [1,2]. This analogy informs a testable mechanistic framework with explicit predictions, measurable variables, and defined boundary conditions. Cellular senescence, inflammation, immune cell recruitment, and extracellular matrix (ECM) remodeling can serve adaptive roles in development, tumor suppression, and tissue repair. When these responses persist, become mistimed or spatially misplaced, or fail to terminate after the initiating insult has been controlled, they can impair regeneration and perpetuate pathology [3,4,5].

Recent experimental studies support specific mechanisms relevant to this framework. For example, reducing signaling through the prostaglandin E2 receptor EP2 in tissue-resident macrophages improved senescent-neutrophil clearance, preserved mitochondrial function, and attenuated selected age-associated organ phenotypes in mice [6]. Defective efferocytosis may allow apoptotic cells and their remnants to persist, sustaining damage-associated signaling [7]. These observations motivate examining not only the abundance of inflammatory mediators but also the progression from damage recognition through debris clearance, immune termination, matrix normalization, and tissue reconstruction to functional recovery. Accordingly, the response to a defined physiological challenge may provide information about resilience that is not captured by a single biomarker measured at rest.

The hallmarks of aging framework organizes interacting age-associated processes into primary, antagonistic, and integrative categories [8,9]. Damage-centered theories describe the generation and accumulation of deleterious changes [10,11]. Evolutionary theories address why natural selection does not ensure indefinite somatic maintenance [12]. Antagonistic pleiotropy theory holds that aging evolves through selection favoring alleles that increase fitness early in life despite detrimental effects on survival or reproduction later in life, while the disposable soma theory links damage accumulation to resource-allocation trade-offs involving growth, reproduction, and somatic maintenance. Geroscience investigates whether targeting shared aging mechanisms can prevent, mitigate, or delay multiple chronic diseases and functional decline [13,14]. The failed-resolution framework occupies an intermediate mechanistic level. It addresses how tissues respond to accumulated damage over time, why protective responses sometimes become self-maintaining, and why damaged tissues increasingly fail to regain structural and functional integrity. Cellular senescence and chronic inflammation can sustain damage-response activity, and the failed-resolution framework examines whether impaired clearance or response termination contributes to their persistence. Autophagy, mitochondrial function, and stem-cell competence influence disposal and reconstruction. These connections are already recognized within the hallmarks framework [9]. The proposed additional contribution is to examine whether the timing and completeness of recovery help explain persistent dysfunction and identify opportunities for intervention.

In this narrative review, we examine whether failed resolution constitutes a coherent, measurable, causally informative, and therapeutically actionable component of human aging. We distinguish analogy from mechanism, define boundary conditions, identify competing explanations, and formulate testable predictions that can support or challenge specific applications of the framework. We then integrate evidence concerning damage-associated signals, nucleic-acid sensing, cellular senescence, immune remodeling, organelle quality control, nutrient sensing, fibrosis, and regenerative exhaustion. We also propose measurements spanning damage burden, danger sensing, resolution competence, regenerative reserve, organ function, and recovery after standardized challenge. Finally, we evaluate candidate interventions while distinguishing mechanistic and animal evidence from human evidence and separating target engagement from clinically meaningful functional benefit [15,16]. The most important question is not whether aging can be reduced to a wound, but whether impaired resolution predicts functional decline, mediates its progression, and can be modified to extend healthy lifespan.

## 2. Conceptual Scope and Testable Predictions of the Failed-Resolution Framework

### 2.1. Tissue Repair as a Regulated Trajectory

Acute tissue repair is a coordinated trajectory rather than a fixed succession of discrete stages. Hemostasis, inflammation, proliferation, angiogenesis, matrix remodeling, and re-differentiation overlap, and their timing varies among tissues [17,18]. Nevertheless, successful repair requires directionality. Damage recognition and host defense must progress toward debris clearance, inflammatory termination, cellular replacement, matrix normalization, and recovery of tissue-specific function. Resolution is therefore an active biological process, not the passive disappearance of inflammatory mediators. It involves cessation of neutrophil recruitment, macrophage efferocytosis, metabolic reprogramming, production of pro-resolving mediators, removal of provisional matrix, and restoration of vascular, barrier, and parenchymal function [19,20]. Histological closure alone is insufficient because it can coexist with fibrosis, impaired mechanics, or incomplete functional recovery.

Cellular senescence illustrates why the timing and context of a damage response matter. Transient senescent states can restrict proliferation, remodel ECM, recruit immune cells, and provide trophic signals that support repair. In mouse skin wounds, CCN1-induced fibroblast senescence limits excessive fibrosis during tissue repair [21,22]. Rapid p21-associated senescence can also arise through translational regulation before extensive transcriptional remodeling, distinguishing wound-associated senescence from slowly accumulated replicative senescence [4]. Dermal adipocyte lipolysis, myofibroblast conversion, and metabolic adaptation provide additional substrates and structural support [23]. The relevant distinction is therefore not simply between senescent and nonsenescent cells. It is between a spatially restricted, functional, and subsequently cleared state and a persistent, heterogeneous state embedded within a dysfunctional immune and stromal environment.

### 2.2. Operational Definition of Failed Resolution

DNA damage, deleterious somatic mutations, epigenetic alterations, chronic inflammation, stress, harmful metabolic by-products, infection, and environmental exposures can contribute to cellular and tissue damage throughout life [9,11]. Such damage can elicit an initially appropriate response. When clearance, cellular replacement, or tissue reconstruction is incomplete, unresolved damage and persistent response activity can alter how the tissue responds to subsequent injury. Age-related changes in skin structure and function illustrate how altered tissue conditions can impair repair [24]. Within this framework, failed resolution can occur in different tissues and across successive episodes of damage.

Failure can occur at multiple transitions. Neutrophils may persist, macrophages may have reduced efferocytic capacity, senescent cells may accumulate and evade immune clearance, fibroblasts may remain activated, and progenitor cells may fail to restore lost parenchyma [7,9,18]. In aged mice, reduced microglial efferocytosis is associated with increased accumulation of apoptotic cells after retinal ischemia/reperfusion injury [25]. In the aged mouse intestine, altered eicosanoid-dependent immune–microbiota interactions impair inflammatory resolution [26]. Senescent cells can impair macrophage clearance of neighboring apoptotic cells through the CD47-QPCT/L axis [27]. In neonatal mice, efferocytosis promotes macrophage arachidonic acid metabolism that supports cardiac regeneration [28]. Reducing PGE2-EP2 signaling in aged mice restores tissue-resident macrophage clearance of senescent neutrophils and improves age-related functional outcomes [6].

**Figure 1:**
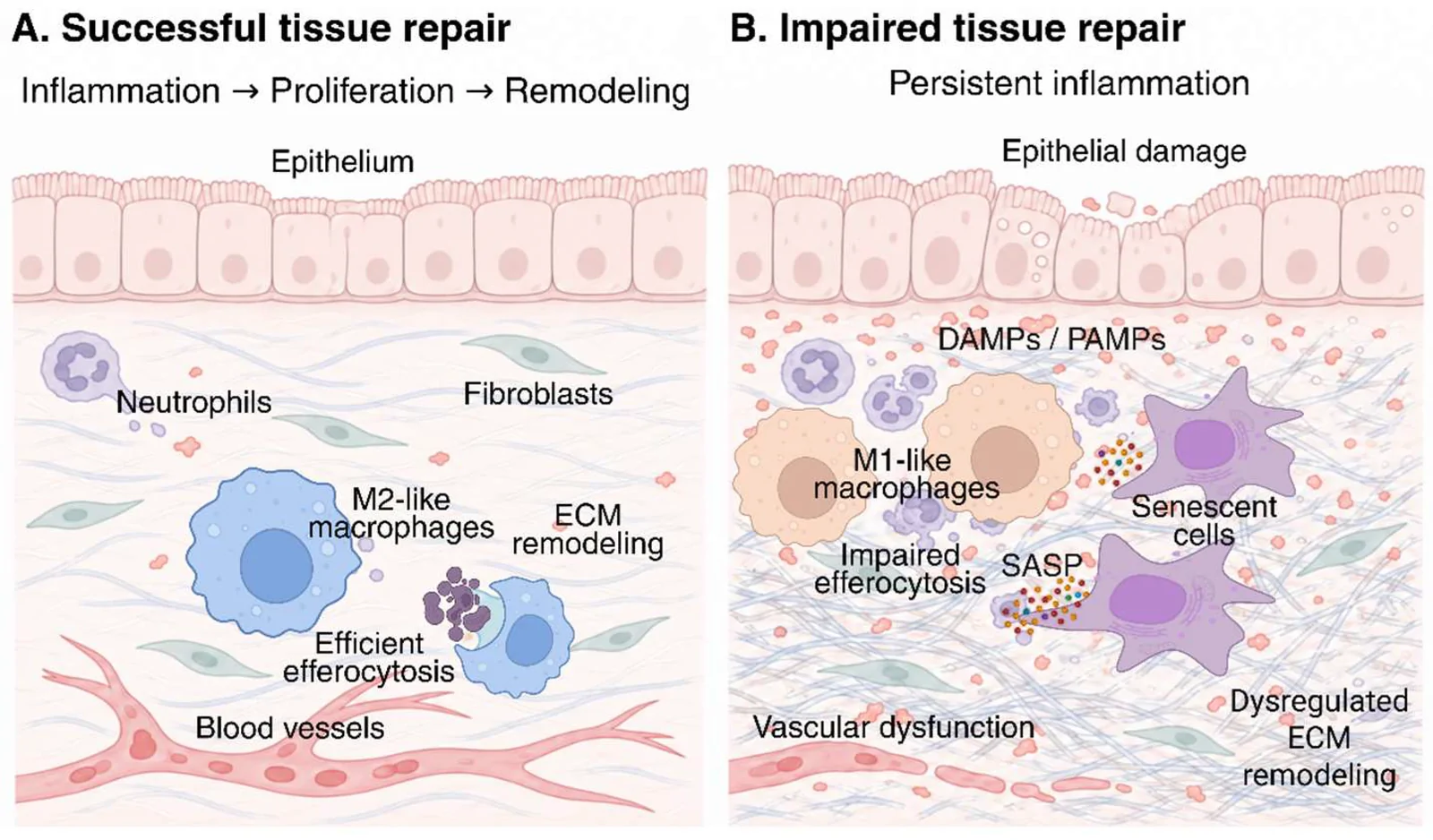
Successful versus impaired tissue repair in aging. (**A**) In successful repair, transient inflammation resolves and gives way to proliferation and remodeling. Neutrophils are cleared, M2-like macrophages remove apoptotic cells by efferocytosis, fibroblasts remodel the extracellular matrix (ECM), and epithelial and vascular integrity is restored. (**B**) In aged tissue, damage- and pathogen-associated molecular patterns (DAMPs and PAMPs, respectively) persist, inflammation fails to resolve, and pro-inflammatory M1-like macrophages predominate. Impaired efferocytosis leaves apoptotic cells uncleared, senescent cells accumulate and secrete senescence-associated secretory phenotype (SASP) factors, ECM remodeling becomes dysregulated, and epithelial and vascular integrity deteriorates. Created with BioRender.com and modified by the authors.

We define failed resolution as a systems-level state in which deficits in damage clearance, response termination, or tissue reconstruction contribute to persistent tissue dysfunction. Resolution capacity encompasses intracellular disposal, efferocytosis, immune termination, vascular and matrix normalization, and cellular replacement. The framework distinguishes three dimensions to be assessed separately: damage input, resolution capacity, and residual functional deficit. Neither low cytokine levels nor the disappearance of histological signs of inflammation establishes successful resolution. Bioenergetic insufficiency can constrain repair, but it is one of several contributing mechanisms [2]. The major transitions from successful repair to age-associated failed resolution are summarized in Table 1, and the resulting tissue states are shown in Figure 1.

### 2.3. Explanatory Scope and Boundary Conditions

The wound analogy has a limited explanatory scope. Human aging develops over decades and affects organs with markedly different regenerative capacities. Although some age-related tissue changes resemble those seen in chronic wounds, aging involves multiple interacting processes that extend beyond incomplete tissue repair. Patterns of inflammaging also vary among individuals and populations [29]. Failed resolution should therefore be treated as a mechanistic model of one important dimension of mammalian aging. This framework can complement molecular-damage theories and the hallmarks of aging. It proposes a proximate explanation for how heterogeneous insults can converge on persistent tissue dysfunction, but it does not by itself explain the evolutionary origin of aging, the primary determinants of damage production, or species differences in somatic maintenance.

Some tissues can deteriorate primarily through cell loss, changes in clonal composition, mechanical failure, or disruption of cell identity. The model is most applicable when incomplete repair contributes to the resulting dysfunction. It should not be used to classify every age-associated abnormality as evidence of failed resolution. For example, replication-associated telomere attrition, stochastic mutation accumulation, chemical modification of long-lived proteins, and mutation-dependent clonal selection can arise independently of failed tissue resolution [9,10,11,30]. The framework applies to their functional consequences only when an independently assessed impairment in clearance, response termination, or tissue reconstruction contributes to those consequences. Their occurrence alone does not establish failed resolution. Persistent dysfunction may reflect excessive or ongoing damage input, impaired clearance, or irreversible loss of cells or tissue architecture. These possibilities are not mutually exclusive. Failure to return to baseline is not, by itself, evidence of a defective resolution mechanism.

### 2.4. Testable Predictions, Falsification, and Causal Evidence

The framework generates five testable predictions. First, functional decline should be preceded or accompanied by persistent damage-response activity and incomplete recovery, rather than merely by elevated inflammatory markers at rest. Second, measures of clearance, remodeling, and recovery kinetics should predict morbidity beyond baseline damage burden and established biological-age measures. Third, restoring clearance or reconstruction should improve tissue function even when the upstream rate of damage generation remains unchanged. Fourth, therapeutic effects should depend on timing because suppressing an early adaptive response may impair repair, whereas correcting its persistence may improve later outcomes. Fifth, organ-specific resolution failures should help explain asynchronous aging among tissues within the same individual.

The model would be weakened if damage-response persistence failed to predict functional decline, if recovery measures added no information beyond established predictors, or if selective restoration of resolution repeatedly failed to improve function despite verified target engagement. Conversely, reduced cytokine concentrations without restored clearance, tissue integrity, or function would not corroborate the model. Shared molecular markers are likewise insufficient because the same mediators can participate in host defense, regeneration, fibrosis, tumor suppression, and tissue injury. Strong mechanistic evidence is strengthened by temporal ordering, cell-type-specific perturbation where feasible, target engagement, mechanistic rescue, and recovery of tissue function. These standards are particularly important for senescence because transient senescent states can be beneficial, available markers lack universal specificity, and susceptibility to senolytic agents varies among cell types [3,4,5,31]. Ultimately, the framework succeeds only if resolution-specific measurements and interventions explain outcomes that damage burden, inflammatory markers, and existing predictors of biological aging do not.

## 3. From Damage to Chronic Dysfunction: Molecular Mechanisms of Failed Resolution

### 3.1. Misplaced Nucleic Acids and Persistent Danger Signaling

Genomic lesions, telomere dysfunction, replication stress, proteotoxicity, mitochondrial defects, and damaged ECM generate signals that normally initiate repair. During aging, altered nuclear architecture, micronuclear rupture, defective DNA repair, mitochondrial membrane permeabilization, and insufficient nuclease activity allow nuclear DNA, mitochondrial DNA (mtDNA), mitochondrial RNA, and retroelement-derived nucleic acids to appear in the cytosol or extracellular space [32,33]. When mislocalized, these endogenous nucleic acids can be recognized as danger signals. Cytosolic double-stranded DNA activates cyclic GMP-AMP synthase (cGAS), which produces cGAMP; cGAMP then activates stimulator of interferon genes (STING). Downstream TBK1-IRF3 and NF-κB signaling induces type I interferons, chemokines, inflammatory cytokines, and components of the senescence-associated secretory phenotype (SASP).

Experiments in cellular models showed that cGAS contributes to senescence-associated inflammatory signaling and that cytoplasmic chromatin fragments can activate cGAS-STING [34,35]. Mitochondria-to-nucleus signaling also promotes cytoplasmic chromatin formation, which connects mitochondrial dysfunction with nuclear inflammatory output [36]. More recent studies identify multiple proximal sources of immunogenic nucleic acids. Apoptotic stress can release mtDNA during senescence and drive the SASP, while defective mitophagy in aged macrophages permits mtDNA leakage and sterile liver inflammation [37,38]. Mitochondrial RNA leakage can similarly sustain the SASP, which broadens the pathway beyond DNA sensing [39]. MRE11 and TREX1 coordinate replication-stress processing and interferon signaling, showing that both production and disposal of nucleic-acid fragments determine the inflammatory state [40].

Intervention studies in naturally aged mice support a causal contribution of this pathway, but not a general causal identity between cGAS-STING activation and aging. Pharmacological or genetic STING inhibition reduces inflammatory transcription in senescent human cells and aged mouse tissues, improves selected measures of physical function, and attenuates microglial activation, neurodegeneration, and cognitive decline [41]. Mitophagy can restrict age-associated cGAS-STING activation by limiting cytosolic mtDNA [42], and ribonucleotide incorporation into mtDNA can promote inflammatory mitochondrial instability [43]. Loss of heterochromatin also permits LINE-1 transcription and reverse transcription, generating cytosolic nucleic acids and type I interferon signaling. Suppressing LINE-1 activity reduces inflammatory phenotypes in experimental systems and alleviates aspects of cardiac aging in mice [44,45]. These findings position misplaced nucleic acids as biomarkers of failed intracellular sanitation and as context-dependent mediators of chronic damage signaling. The extent to which they contribute to human aging is not yet established.

### 3.2. Chronic Inflammation, Inflammasomes, and Inflammatory Amplification

The NLRP3 inflammasome integrates mitochondrial ROS, ion flux, lysosomal damage, extracellular ATP, protein aggregates, and nucleic-acid stress. Assembly of NLRP3 with ASC and pro-caspase-1 generates active caspase-1, mature IL-1β and IL-18, and, in some contexts, gasdermin-D-dependent pyroptosis. An age-associated reduction in SIRT2-dependent NLRP3 deacetylation increases inflammasome activity and contributes to chronic inflammation and insulin resistance in mice [46]. NLRP3 activation can also reinforce senescence, endothelial dysfunction, fibrosis, and stem-cell impairment. Pharmacological inhibitors such as MCC950 and dapansutrile demonstrate targetability in experimental disease, but long-term inhibition in otherwise healthy older adults requires careful evaluation because inflammasomes contribute to antimicrobial defense and tissue repair [47,48].

NF-κB integrates signals from Toll-like receptors, cGAS–STING, and cytokine receptors and contributes to inflammasome priming [33,47,48]. Persistent NF-κB activity maintains SASP factors, chemokines, adhesion molecules, and procoagulant tissue factor. The coagulation and complement systems, in turn, amplify inflammation through thrombin, protease-activated receptors, platelet activation, endothelial adhesion, and complement-derived anaphylatoxins. This coupling helps explain why age-related sterile inflammation is associated with a prothrombotic state and why cell-free DNA and damaged endothelium may be both biomarkers and effectors. It also cautions that anti-inflammatory treatment alone may not restore resolution if efferocytosis, matrix remodeling, and vascular repair are not restored.

### 3.3. Cellular Senescence as a Context-Dependent Response to Various Stresses

Cellular senescence comprises durable proliferative arrest accompanied by variable changes in chromatin, metabolism, lysosomes, morphology, resistance to apoptosis, and secretion [49,50]. No single marker identifies all senescent cells in vivo. p16INK4A, p21CIP1, senescence-associated beta-galactosidase, loss of lamin B1, DNA-damage foci, and SASP components must be interpreted as a composite and within tissue context [51,52]. Current in vivo guidelines recommend p16INK4A and/or p21CIP1 together with independent auxiliary markers, defined cell identity, appropriate controls, and tissue-specific interpretation [31]. Senescence-associated markers can also emerge before stable cell-cycle arrest or occur in nonsenescent cells, and lysosomal beta-galactosidase activity can be elevated in macrophages [31,53]. Absent proliferation alone does not establish senescence in postmitotic cells. Transcriptomic signatures improve classification, but their performance depends on the inducing stress and cell lineage [54]. Recent evidence further indicates that some senescent states may escape arrest or change phenotype, which challenges a binary irreversible-versus-reversible definition [55].

Senescence supports embryogenesis, wound repair, tumor suppression, and tissue remodeling when it is spatially restricted and followed by immune clearance [22,56]. Harm arises when production exceeds removal or when the SASP becomes chronic. IL-6, IL-1 family cytokines, CXCL chemokines, TGF-β, matrix metalloproteinases, growth factors, and extracellular vesicles can induce paracrine senescence, recruit myeloid cells, disrupt stem-cell niches, and promote fibrosis. Hypoxia and energy status modify this output. Physiological hypoxia activates AMPK and suppresses mTOR-dependent SASP production, which demonstrates that secretory activity can be uncoupled from arrest [57]. The cGAS-STING-PERK axis also supports a translation-dependent response required for senescence and fibrosis, linking innate sensing to protein synthesis [58].

The pathological unit is therefore not merely a p16-positive cell. It is a multicellular circuit involving senescent parenchymal and stromal cells, immune surveillance, matrix, vasculature, and neighboring progenitors. Senescent alveolar type 2 cells can drive progressive pulmonary fibrosis, and senescence can prevent renal regeneration after injury [59,60]. Whole-body senescent-cell clearance can reduce brain inflammation in mice, while post-sepsis liver studies show tissue-specific senescence states and responses to senolytics [61,62]. In the aged liver, p21+TREM2+ macrophages have been implicated in inflammaging and metabolic dysfunction-associated steatotic liver disease [63]. Human skeletal-muscle multiomics reveals senescence-associated cell states and nominates CCR5 antagonism as a possible senotherapeutic strategy, but clinical efficacy has not been established [64]. These examples underscore senescent-cell heterogeneity and support organ- and cell-state-aware targeting rather than assuming uniform susceptibility.

### 3.4. Immune Remodeling and Defective Clearance

Immune aging combines quantitative and qualitative changes in hematopoietic stem cells, myeloid bias, thymic involution, loss of naive lymphocytes, restricted receptor diversity, impaired vaccine responses, chronic innate activation, and altered tissue-resident macrophages [65]. In mice, an aged immune system can induce senescence and organ damage, supporting bidirectional interactions between immune remodeling and senescent-cell accumulation [66]. Inflammatory exposure leaves durable epigenetic and metabolic effects in hematopoietic stem cells, reducing self-renewal and accelerating myeloid-biased aging [67]. Somatic mutations in DNMT3A, TET2, ASXL1, JAK2, and related genes can confer a clonal growth advantage. Clonal hematopoiesis is associated with increased risks of cardiovascular disease, hematologic malignancy, and mortality, with risk varying by mutation and clone size [30]. Resolution depends on competent phagocytes. Aging impairs chemotaxis, phagocytosis, efferocytosis, mitochondrial metabolism, and the transition from inflammatory to reparative macrophage states [7,68]. Senescent neutrophils and cellular remnants can persist, release proteases and oxidants, and maintain complement activity. Conditional EP2 deletion initiated in young adulthood attenuated multiple age-associated organ phenotypes assessed in aged mice. Pharmacological EP2 inhibition restored macrophage clearance of senescent neutrophils in aged mice [6]. Genetic impairment of perforin-dependent immune surveillance accelerated senescent-cell accumulation, multiorgan fibrosis, and mortality in mice, implicating clearance capacity more broadly than macrophage efferocytosis alone [69]. Senescent cells can also actively resist removal, including through inhibitory immune-checkpoint ligands, which distinguishes loss of clearance capacity from evasion by the target-cell [70]. Together, these findings support clearance failure as a causal contributor to selected organ phenotypes in mice rather than as a demonstrated general mechanism of human aging. Specialized pro-resolving mediators, including resolvins, protectins, maresins, and lipoxins, can restrict neutrophil recruitment and enhance macrophage efferocytosis without broadly suppressing host defense. Evidence is strongest in inflammatory, cardiac-repair, and vascular models, while pharmacokinetics, receptor specificity, and appropriate clinical endpoints have yet to be adequately defined [20,71].

Human mechanistic evidence is available from cantharidin-induced skin inflammation. Older participants showed impaired macrophage efferocytosis associated with reduced TIM-4 expression and elevated p38 MAPK activity. Oral p38 inhibition restored TIM-4 expression, reduced retained apoptotic material, and improved the macrophage resolution phenotype [72]. Losmapimod improved local inflammatory resolution without detectable changes in 24 h blister TNF-α, IL-6, or neutrophil counts [72]. These findings support the framework but do not directly test its third prediction. Because treatment began before cantharidin application, the study did not test treatment initiated after the induced inflammatory response was established. This supports reversibility of a local resolution defect, not modification of systemic human aging. Together with clearance-restoration experiments in aged mice [6], it supports a context-specific causal contribution to tissue dysfunction.

### 3.5. Mitochondrial and Lysosomal Constraints on Repair

Repair requires energy, redox control, biosynthetic substrates, and metabolic flexibility. Cell migration, phagocytosis, matrix remodeling, angiogenesis, and progenitor proliferation depend on coordinated ATP production and substrate allocation. Aging mitochondria show altered dynamics, reduced respiratory reserve, mtDNA lesions, impaired electron transport, and disturbed communication with lysosomes, endoplasmic reticulum, and nucleus [73,74,75]. These changes affect ROS signaling, calcium handling, one-carbon metabolism, acetyl-CoA availability, innate immunity, and cell fate. However, mitochondrial suppression can sometimes be adaptive during acute stress, and glycolysis can support effective immune defense and repair. Therefore, the decisive defect is not simply low oxidative phosphorylation. It is loss of the capacity to switch metabolic programs appropriately and then return to a state compatible with tissue reconstruction.

Autophagy and mitophagy remove damaged proteins, organelles, and intracellular debris, thereby reducing the signals that initiate sterile inflammation. Autophagic capacity declines in multiple aging tissues, and defective flux can coexist with increased autophagosome markers, which makes static measurements difficult to interpret [76]. In aged hematopoietic stem cells, autophagy counteracts inflammation-driven glycolytic impairment, while correction of lysosomal dysfunction can restore a more youthful stem-cell state [77,78]. Suppressed basal mitophagy can produce cellular aging phenotypes that are reversible with a p62-directed small molecule in experimental systems [79]. These findings support enhancement of quality-control flux, but excessive autophagy can be harmful and tissue-specific dose–response relationships require definition.

NAD+ couples metabolism, DNA repair, chromatin regulation, and stress responses through sirtuins, PARPs, and other NAD-consuming enzymes. NAD+ availability declines in several, although not all, aged tissues and experimental contexts, partly through induction of CD38 in inflammatory immune cells [80,81]. Reduced NAD+ may compromise mitochondrial and repair functions, while inflammatory activation can further increase consumption. mTORC1 promotes growth and protein synthesis when nutrients and mitogens are sufficient, whereas AMPK restrains anabolic demand and supports catabolic maintenance during energy stress. Chronic or mistimed mTORC1 activity may maintain growth and secretion after damage, but continuous suppression can impair immunity, wound closure, and regeneration. The therapeutic objective is restoration of context-sensitive metabolic switching rather than permanent activation of catabolism or indiscriminate inhibition of growth [82].

### 3.6. Extracellular Matrix Remodeling and Fibrosis

The ECM is not merely a structural scaffold. Its composition, architecture, matrix-bound growth factors, and mechanical properties regulate cell behavior through integrins, focal adhesion kinase, YAP/TAZ, TGF-β, and related mechanotransduction pathways. Aging alters this regulatory environment through collagen fragmentation and cross-linking, elastin deterioration, changes in glycosaminoglycans and matrix-bound signals, and increased or spatially heterogeneous stiffness. These changes can impair cell migration and vascular exchange, distort stem-cell niches, and favor persistent myofibroblast activation [83]. Matrix stiffening can also induce chondrocyte senescence through epigenetic mechanisms [84]. Thus, damaged ECM is not only an endpoint of tissue aging. It can become a source of signals that perpetuate cellular dysfunction and further compromise repair.

Fibrosis represents an advanced form of failed remodeling. Persistent TGF-β signaling, survival of activated myofibroblasts, inflammatory macrophage states, senescent epithelial cells, and cross-linked matrix can progressively replace specialized parenchyma with mechanically abnormal scar tissue. The signaling involved is strongly context-dependent. In some stromal settings, preserved YAP/TAZ activity may limit aging-associated phenotypes by restraining cGAS-STING signaling. This protective stromal effect should not be generalized, because YAP/TAZ functions vary by cell type, tissue, and disease context [85]. Studies in liver and kidney models further implicate BAZ2B-dependent chromatin regulation and macrophage iron handling in the coupling between senescence and fibrosis [86,87]. These findings place matrix organization, vascular function, and cell-state regulation within the same resolution problem. Regeneration is unlikely to restore durable tissue function if new or surviving cells remain embedded in a stiff, poorly perfused, and profibrotic niche.

### 3.7. Regenerative Exhaustion and Asynchronous Organ Aging

Stem-cell exhaustion is not a simple depletion of cell number. It includes altered quiescence, metabolic rigidity, DNA damage, epigenetic drift, inflammatory memory, defective adhesion, clonal selection, and changes in niche signals. Single-cell comparisons across tissues show distinct trajectories of regenerative decline rather than one uniform stem-cell aging trajectory [88]. Hair-follicle stem cells can physically escape their niche during aging, while chronic inflammatory exposure produces long-lived defects in hematopoietic stem-cell self-renewal [67,89]. Recent work in muscle proposes that selective survival of particular stem-cell states can itself distort the population and drive functional aging [90].

Because exposure, damage production, repair capacity, and tissue architecture differ among organs, biological aging is asynchronous. Vascular dysfunction can compromise perfusion across kidney, brain, and muscle, while immune aging can alter surveillance and inflammatory tone in every tissue. Liver and kidney influence systemic metabolite clearance, and the gut barrier and microbiota shape immune activation. Recent cross-sectional histopathology-based analysis of approximately 1000 human donors identified distinct, often nonlinear structural aging trajectories across tissues, with partially coordinated periods of acceleration [91]. This evidence strengthens the case for organ-specific phenotyping while cautioning against treating a whole-body score as a literal measure of every tissue. A clinically useful assessment should quantify shared systemic processes, organ-specific failure modes, and the coupling between them.

## 4. Biomarkers

### 4.1. Biological-Age Biomarkers

Biological-age biomarkers are commonly trained to predict chronological age, mortality, disease, or functional decline [92]. Predictive accuracy does not establish mechanism, and a clock can change without the targeted aging process changing. The failed-resolution framework instead specifies six complementary domains: damage burden, danger-sensing activity, resolution competence, regenerative reserve, organ structure and function, and recovery dynamics. Damage markers may include somatic mutation burden, DNA-damage responses, cell-free nuclear and mitochondrial DNA, oxidized macromolecules, protein aggregates, and matrix fragments. Activation markers may include interferon-stimulated genes, IL-1β, IL-6, TNF, CRP, SASP panels, inflammasome products, complement, and coagulation signals. Resolution assays should quantify efferocytosis, neutrophil clearance, macrophage state transitions, specialized pro-resolving mediators, and decay of inflammatory signals after challenge.

Regenerative measures need to assess vascular responses, matrix turnover, stem or progenitor competence, and tissue-specific repair. Functional outcomes should include strength, mobility, cognition, immune competence, and organ reserve. This architecture prevents a common inferential error, namely interpreting lower inflammation as restored health. A drug may suppress IL-6 while impairing pathogen defense, wound closure, or regenerative signaling. Conversely, an acute inflammatory rise after vaccination or exercise may be an appropriate response. The relevant variables are response amplitude, localization, timing, decay, recovery completeness, and functional consequence. Immune-aging biomarker recommendations for clinical trials accordingly emphasize multidimensional immune function and challenge-response assays rather than resting cell counts alone [93]. Composite indices should retain their component domains, because the same total score can arise from biologically different and therapeutically opposite profiles.

### 4.2. Epigenetic, Proteomic, Metabolomic, and Organ-Specific Clocks

DNA-methylation clocks have established that reproducible age-associated molecular patterns can estimate chronological and phenotypic age [94,95]. GrimAge incorporates methylation surrogates for plasma proteins and smoking history and predicts mortality and healthspan-related outcomes [96]. DunedinPACE estimates the longitudinal pace of aging from blood methylation and is suited to intervention studies, although technical reliability, population transferability, and within-person interpretation are important limitations [97,98]. Stress-associated increases in epigenetic and multiomic biological age can reverse after recovery, which supports dynamic measurement but also shows that clock changes may reflect transient physiology rather than durable rejuvenation [99].

Proteomic, metabolomic, immune, imaging, histopathologic, and routine-clinical clocks add complementary information. Multi-tissue proteomics reveals organ-specific trajectories and plasma signatures linked to tissue sources [100,101]. Metabolome-based age gaps associate with cardiometabolic disease and mortality, while multi-organ MRI clocks identify organ-specific and cross-organ relations with disease and cognition [102,103]. Single-cell immune clocks can map heterogeneous T-cell aging [104]. Routine chemistry and organ-function data may improve clinical scalability [105,106]. However, organ-specific clocks are not interchangeable [107,108]. Systems Age and related approaches attempt to recover within-person heterogeneity from blood, while histopathology-based structural aging directly quantifies tissue architecture [91,109]. A 2026 cross-species analysis identified conserved transcriptomic signatures, including CDKN1A and LGALS3, whose protein concentrations were associated with multimorbidity and mortality [110]. These findings support biomarker discovery but do not establish causal specificity or surrogate validity.

Current multiomic studies increasingly show that people reach similar global scores through different molecular routes. Proteomic aging phenotypes, organ-level clocks, and cross-organ analyses identify substantial heterogeneity and dependence among tissues [111,112,113,114]. This heterogeneity is clinically useful if it guides intervention selection. It becomes a problem when composite scores conceal opposing changes, such as improved inflammation with worsened renal function. For failed-resolution studies, clocks should be accompanied by pathway-specific markers and direct functional endpoints.

### 4.3. Validation Requirements

A biomarker intended as a surrogate endpoint must demonstrate analytical validity, biological interpretability, association with meaningful outcomes, responsiveness to intervention, and evidence that biomarker change predicts benefit rather than harm [115,116]. Age prediction is insufficient. Validation should address ancestry, geography, sex, socioeconomic conditions, multimorbidity, medication, and preanalytic handling. External cohorts, calibration, measurement error, batch effects, missingness, and prespecified analysis plans are essential. For dynamic markers, reproducibility includes the challenge protocol, sampling schedule, kinetic model, and test–retest reliability. For composite scores, investigators should report domain-level changes so that improvement in one component cannot conceal deterioration in another.

Intervention studies need to prespecify whether each biomarker represents state, rate, response, or recovery. Caloric restriction changes some methylation measures while leaving others unchanged, and telomere findings differ from pace-of-aging scores [117,118]. A credible geroscience endpoint should pair a validated molecular measure with function or clinical outcome. Biomarkers may shorten trials, but they cannot yet substitute for improved healthspan, delayed multimorbidity, preserved independence, or reduced mortality [119]. Causal mediation analysis can test whether change in a proposed resolution pathway statistically accounts for functional benefit, but this approach is vulnerable to measurement error, time-varying confounding, and incorrect causal models.

### 4.4. Operationalizing a Resolution Phenotype

A resolution phenotype should be estimated as a profile, not assumed from one marker. At minimum, studies should report baseline damage burden, peak response, clearance or decay kinetics, recovery completeness, and tissue function. A useful analysis can model each participant as a trajectory through a state space defined by damage-response activity and functional reserve. Successful recovery moves the system back toward low residual damage-response activity with preserved function. The framework predicts that some age-related resolution defects will delay recovery and leave a larger residual displacement after stress. Comparison across challenges requires appropriately standardized variables and challenge-specific interpretation.

Prospective validation requires three steps. First, demonstrate analytical reproducibility and short-term within-person stability under standardized conditions. Second, show that the dynamic phenotype predicts future functional decline or multimorbidity after adjustment for age, sex, baseline disease, resting biomarkers, and established clocks. Third, test whether an intervention changes the prespecified trajectory and whether that change mediates functional benefit. The 2026 cross-species damage-production and removal model provides a mathematical precedent for separating damage input from removal capacity, although its species-level regimes require direct experimental validation in human tissues [120].

### 4.5. Candidate Human Research Protocols

No validated clinical test currently quantifies a patient’s overall resolution competence. The following measurements are proposed research endpoints, not diagnostic criteria or established surrogate outcomes. Human cantharidin-blister studies provide a local model in which separate blisters can be sampled at 24 and 72 h to compare leukocyte and mediator profiles [121]. This approach has also revealed age-associated, pharmacologically modifiable efferocytic defects [72]. Additional sampling times would be needed to identify the true response peak and estimate a resolution interval. A decline between two time points is not a direct measure of efferocytosis, because cell influx, death, and egress also affect leukocyte counts.

A practical pilot could combine local challenge sampling with paired blood collection and a standardized, clinically appropriate functional challenge. The six domains below should be analyzed separately before any composite score is constructed. Where tissue is sampled, collection from separate matched sites avoids disturbing the same lesion repeatedly. Repeated prechallenge measurements, blinded assays, standardized handling, and prespecified adjustment for comorbidity and medication are required. The schedule and readouts are proposals for validation, not protocols established by the cited studies [93,115,116]. Candidate measurements across the six domains are summarized in Table 2.

**Table 2:** Candidate human research measurements across six biomarker domains.

Domain	Candidate Measurements in Humans	Interpretive Constraint
Damage burden	Blood before and after challenge, with local exudate during the response, for cell-free DNA and matrix fragments	Concentrations reflect release and systemic clearance. They do not identify a specific tissue defect
Danger sensing	Serial cytokines and interferon-response transcripts paired with local leukocyte counts	Specify peak, postpeak trajectory, and residual response. Lower values can reflect suppression of an appropriate response
Resolution competence	Blister-cell analysis plus ex vivo uptake of labeled apoptotic targets by recovered phagocytes. Confirm internalization by imaging or extracellular-signal exclusion	Report target apoptosis, phagocyte viability, target-to-cell ratio, incubation time, uptake inhibition controls, and digestion separately
Regenerative reserve	In a suitable skin-repair substudy, serial imaging of epithelial closure and barrier recovery, with perfusion and matrix measurements	Accessible-tissue repair cannot establish regenerative capacity in brain, heart, or other organs
Organ function	Prespecified measures of mobility, strength, or the relevant organ’s function before challenge and after recovery	Interpret change against measurement error and clinically meaningful thresholds
Recovery dynamics	After an individualized exercise challenge, for example, repeat symptom assessments and performance measurements, at 24 and 48 h, and until recovery	This is an illustrative schedule. Match workload to capacity, allow longer follow-up, and test reproducibility. Exercise recovery is not specific to inflammatory resolution

## 5. Candidate Interventions to Restore Resolution While Preserving Repair

### 5.1. Therapeutic Principles and Timing

The failed-resolution framework changes the intervention question from identifying one pathway that causes aging to identifying the dominant, measurable failure in a particular tissue and time window. Excess damage production may require exposure reduction or molecular maintenance. Persistent danger sensing may justify temporary, biomarker-selected inhibition of cGAS-STING, NLRP3, JAK-STAT, or related pathways. Senescent-cell accumulation may require selective removal or phenotype modulation. Clearance failure may require restoration of macrophage metabolism, efferocytosis, and pro-resolving signaling. Regenerative failure may require vascular, matrix, progenitor, or epigenetic intervention. Combination therapy is mechanistically plausible because these defects reinforce one another, but it also can increase toxicity and complicate causal attribution. Candidate treatments should be ranked by reproducibility, target engagement, evidence level, effect size, durability, and human safety rather than by isolated changes in lifespan or a molecular clock [122].

Timing is a core part of the mechanism. Depending on the intervention and tissue, treatment after early repair responses may help limit persistent fibrosis. Intermittent senolytic dosing may limit cumulative toxicity, and mTOR inhibitors may require intermittent or tissue-selective schedules. Pro-resolving mediators aim to terminate inflammation while preserving host defense, but this advantage must be demonstrated for each agent and indication. Trials should distinguish prevention of damage, interruption of persistent response, restoration of clearance, and induction of regeneration. Lower inflammatory markers alone do not demonstrate restored resolution, particularly when treatment compromises host defense, tissue repair, or organ function. The principal failure modes and corresponding intervention strategies are summarized in Table 3.

### 5.2. Senolytics and Senomorphics

Genetic clearance of p16INK4A-positive cells delayed age-associated disorders in progeroid mice, and pharmacological senolytics later improved physical function and survival in naturally aged mice [123,124]. Dasatinib plus quercetin, navitoclax, fisetin, HSP90 inhibitors, cardiac glycosides, and related compounds exploit senescent-cell dependencies on pro-survival pathways [125,126,127]. Fisetin improved selected healthspan measures and lifespan in mice, but human tissue exposure, senolytic specificity, and clinical efficacy have not been adequately characterized [128]. Senomorphics instead reduce harmful secretion or modify phenotype without killing the cell. Candidate targets include mTOR, p38 MAPK, JAK, NF-κB, BRD4, cGAS-STING, and specific SASP components [129,130].

Early open-label studies of intermittent dasatinib plus quercetin enrolled 14 participants with idiopathic pulmonary fibrosis and nine with diabetic kidney disease. They reported feasibility and changes in physical performance or tissue senescence markers, respectively, but lacked placebo controls and did not establish broad clinical efficacy [131,132]. Heterogeneity of senescent cells is a major biological obstacle. A surface marker or survival pathway expressed by one senescent cell type may be absent from another and may be shared with essential normal cells. New circulating and tissue markers, including IL-23R-associated signatures, may improve patient selection and pharmacodynamic assessment [133]. A subsequent randomized, single-blind, placebo-controlled phase 1 trial in idiopathic pulmonary fibrosis enrolled 12 participants and likewise addressed feasibility and tolerability rather than clinical efficacy [134].

Immune-based approaches enable antigen-directed targeting but introduce new risks. uPAR-directed CAR T cells produced durable improvements in exercise capacity and metabolism in aged mice, and in vivo CAR engineering may simplify delivery [135,136]. Senolytic vaccination has improved age-related phenotypes in experimental models [137]. These strategies have been evaluated only preclinically in the context of aging, and immune toxicity, antigen escape, impaired repair, autoimmunity, manufacturing, and reversibility require extensive study. Natural extracts with senomorphic activity can improve mouse outcomes, but uncertain composition and pleiotropic targets limit mechanistic attribution [138].

### 5.3. Inhibiting Danger Sensing and Promoting Resolution

The cGAS-STING pathway is attractive because it connects misplaced DNA with type I interferon signaling, SASP production, and neuroinflammation. Small-molecule STING inhibitors improved inflammatory and functional outcomes in aged mice [41]. NLRP3 inhibitors and downstream IL-1 blockade may interrupt inflammasome-driven amplification. However, both pathways participate in host defense, and cGAS-STING also contributes to tumor immune surveillance [46,47,48]. Long-term systemic inhibition could be hazardous, particularly in older adults with immunosenescence. Short courses, tissue-targeted delivery, or biomarker-selected use during persistent sterile activation may offer a better therapeutic index.

Restoring efferocytosis may address a more proximal defect. Candidate approaches include enhancing MerTK signaling or TIM-4-dependent apoptotic-cell recognition, countering CD47-mediated “do not eat me” signals, improving macrophage mitochondrial metabolism, and administering stable pro-resolving mediators. Experimental data show that restoring macrophage clearance can attenuate age-associated organ phenotypes in mice [6], and that resolvin pathways can support vascular resolution [20]. Clinical translation will require assays that distinguish clearance of apoptotic material from inappropriate engulfment of viable cells. It will also require proof that pro-resolving treatment restores tissue function rather than merely lowering cytokines.

### 5.4. Nutrient Sensing: mTOR Inhibition and AMPK-Directed Treatment

Rapamycin is among the most consistently lifespan-extending pharmacological interventions in mice. Late-life treatment extends lifespan in genetically heterogeneous mice [139]. In older humans, everolimus or related TORC1 inhibitors improved influenza-vaccine responses and reduced some infection outcomes in early studies [140,141]. mTOR inhibition can enhance autophagy, reduce SASP translation, restrain inappropriate growth, and improve immune function. Yet rapamycin and rapalogs can cause stomatitis, dyslipidemia, glucose intolerance, cytopenias, edema, infection risk, and impaired wound healing. Dose, schedule, mTORC1 selectivity, tissue distribution, sex, and baseline immune state are therefore decisive [142,143]. Later results were less consistent. In a phase 3 trial of 1024 older adults, RTB101 did not reduce clinically symptomatic respiratory illness (26% versus 25%, odds ratio 1.07, 95% CI 0.80–1.42), although interferon-induced antiviral genes were upregulated in both the phase 2b and phase 3 trials [144]. The two trials differed in primary endpoint, laboratory-confirmed infection versus symptom-based illness, and in enrolled population, because the phase 3 trial excluded current smokers and patients with chronic obstructive pulmonary disease [144,145]. These trials distinguish pathway engagement from clinical benefit. Differences in endpoints and enrolled populations may have contributed to the divergent results, but their contribution remains uncertain [144,145]. These results do not establish that impaired repair caused the negative phase 3 result.

Combination treatment may produce greater efficacy but also greater uncertainty. Trametinib plus rapamycin additively extended mouse lifespan and reduced inflammation and tumors, which supports targeting connected nutrient-sensing and mitogenic pathways [146]. This result is preclinical and cannot justify preventive MEK inhibition in healthy people because trametinib has substantial toxicity. A candidate translational strategy is intermittent, low-dose, or tissue-selective mTORC1 inhibition combined with mechanistic biomarkers and close monitoring of immunity, metabolism, and repair.

Metformin activates AMPK indirectly through energetic stress, inhibits hepatic gluconeogenesis, affects mitochondrial complex I and lysosomal signaling, and can enhance autophagy. It attenuates several hallmarks in experimental models and reduces inflammatory signaling in some contexts [147,148,149,150]. A 40-month study in male cynomolgus monkeys reported slower multiomic aging signatures and neuroprotective effects [151]. Human evidence for treating aging is inconclusive. Observational comparisons are vulnerable to confounding, and trials in people without diabetes have produced mixed molecular and functional results. Recent critical assessment has therefore increased uncertainty about a generalized anti-aging effect [152]. Metformin should be tested as a candidate gerotherapeutic, not described as an established treatment for aging.

### 5.5. NAD+ Restoration and Sirtuin Biology

Nicotinamide riboside (NR), nicotinamide mononucleotide (NMN), nicotinic acid derivatives, CD38 inhibition, and reduced PARP overactivation can raise or preserve NAD+. Human NMN trials demonstrate target engagement and generally acceptable short-term safety. Some studies report improved muscle insulin sensitivity, walking speed, or selected metabolic measures, whereas others find limited functional benefit [153,154,155,156]. In COPD, NR increased blood NAD+ and reduced sputum IL-8 in a small randomized trial, but systemic IL-6 did not change, and the clinical significance is unclear [157]. Systematic review of preclinical and clinical evidence indicates a consistent difference between biochemical efficacy and healthspan efficacy. NR and NMN usually increase NAD-related metabolites, but effects on physical, vascular, cognitive, or metabolic outcomes are heterogeneous and often endpoint-specific [158]. Long-term safety, tissue delivery, dose–response relations, baseline NAD status, methyl-donor demand, tumor biology, and interindividual metabolism require further study [159]. Sirtuin biology is more complex than activation of a single longevity switch. SIRT1, SIRT3, SIRT6, and other family members differ in their substrates, subcellular localization, and physiological effects. Their activity depends on NAD+ availability and varies across cell types, tissues, stress states, and time. Compounds described as sirtuin activators may also act through sirtuin-independent mechanisms. A more defensible therapeutic aim is therefore to restore context-appropriate NAD+-dependent regulation and metabolic flexibility rather than assume that activating one sirtuin, or administering a single supplement, can reproduce the effects of caloric restriction.

### 5.6. Autophagy, Mitophagy, Spermidine, and Urolithin A

Spermidine promotes autophagy partly through eIF5A hypusination and is required for several fasting-associated benefits across experimental organisms [160,161,162]. Dietary spermidine improves cognition in animal models [163], while a randomized trial in older adults with subjective cognitive decline found no significant benefit on its primary memory outcome [164]. These findings support biological activity but also illustrate the gap between mechanistic plausibility and clinically meaningful effect. Urolithin A, a gut-microbial metabolite derived from ellagitannins, induces mitophagy and improves mitochondrial measures. Randomized trials reported improvements in selected secondary muscle outcomes and mitochondrial biomarkers, but not in their primary endpoints: peak power output in middle-aged adults, and six-minute walk distance and maximal ATP production in hand muscles in older adults [165,166]. A recent trial found shifts toward less terminally exhausted CD8+ T cells and improved immune-cell mitochondrial features after four weeks [167]. Larger and longer trials must determine whether these changes reduce infection, frailty, or disability. Baseline microbiome function, diet, mitochondrial phenotype, and physical activity may modify response.

### 5.7. Stem Cells, Extracellular Vesicles, and Regenerative Medicine

Mesenchymal stromal cells can modulate inflammation, angiogenesis, matrix remodeling, and endogenous progenitor activity primarily through paracrine signals rather than durable engraftment. Their extracellular vesicles may deliver proteins, lipids, and regulatory RNAs, but product heterogeneity, potency assays, biodistribution, thrombosis risk, immune compatibility, and manufacturing are major barriers [168,169]. Reviews of stem-cell approaches to aging emphasize that evidence is insufficient for unregulated commercial “rejuvenation” procedures [170]. Controlled trials are beginning to provide more informative evidence. A randomized phase I/II study of umbilical-cord tissue-derived mesenchymal stromal cells in frailty reported acceptable short-term safety and improvement in selected quality-of-life and physical outcomes [171]. A phase 2b trial of laromestrocel found a significant dose–response relationship for six-minute walk distance at six months, although individual dose-versus-placebo comparisons were not significant at that time. Improvements in selected dose groups were significant at nine months, and soluble TIE2 was identified as an exploratory response biomarker [172]. These results are encouraging for frailty treatment, but they do not establish systemic rejuvenation or lifespan extension. Replication, durability, hard clinical endpoints, product comparability, and long-term safety are required. Regeneration also depends on the receiving niche. Cell therapy may fail unless excessive matrix stiffness, vascular insufficiency, senescent-cell burden, and inflammatory signaling are reduced. Sequential treatment could therefore be rational: reduce persistent damage and senescence, restore matrix and vascular conditions, and then support endogenous or transplanted progenitors. This sequence should be tested rather than assumed.

### 5.8. Partial Epigenetic Resetting

Transient expression of OCT4, SOX2, KLF4, with or without MYC, can reverse selected epigenetic and functional features of aging without inducing full pluripotency. Cyclic partial epigenetic resetting improved age-associated phenotypes in progeroid mice, and OSK expression was reported to improve selected epigenetic features and visual function in mouse models [173,174]. Mouse studies report molecular changes consistent with rejuvenation during physiological aging [175]. Targeting OSK expression to Cdkn2a-positive cells improved health-related markers and survival in progeroid models and altered age-associated states in naturally aged mice [176]. The approach is experimental. Incomplete control can cause loss of cell identity, dysplasia, teratoma formation, or cancer. Epigenetic-resetting effects differ by tissue, delivery system, factor combination, duration, and baseline damage. Recent evidence that partial epigenetic resetting can reverse widespread mesenchymal drift provides a possible mechanistic explanation, but its relevance to safe human treatment is unproven [177]. Priorities include nonintegrating delivery, inducible and self-limiting systems, cell-type specificity, exclusion of premalignant clones, and long-term surveillance [178]. A first-in-human phase 1 study of ER-100, an OSK-based epigenetic therapy, has begun in patients with optic neuropathies to evaluate safety and tolerability [179].

### 5.9. Comparative Maturity of Evidence

Among mTOR inhibition, NAD+ supplementation, and partial epigenetic reprogramming, mTOR-directed treatment has the strongest combined basis in mouse longevity studies and human randomized trials, although human clinical results are inconsistent and preventive long-term safety is unresolved [139,140,141,142,143,144]. NAD+ precursors show biochemical target engagement with variable functional benefit [153,154,155,156,157,158,159]. Partial epigenetic resetting has substantial experimental potential but unproven human efficacy and major delivery, cell-identity, and tumor-safety constraints [173,174,175,176,177,178]. There are no direct comparative trials establishing a preferred intervention for human aging or resolution failure.

### 5.10. Lifestyle Interventions, Geroprophylaxis, and Personalized Geroscience

Lifestyle measures provide the best-established basis for geroprophylaxis, understood here as the prevention or delay of age-related disease and functional decline. Exercise and appropriate energy intake influence several processes relevant to failed resolution, including mitochondrial turnover, insulin sensitivity, vascular function, immune regulation, autophagy, and inflammatory activity. Dietary restriction should not, however, be confused with malnutrition, frailty, or loss of lean mass, particularly in older adults [180].

Human studies have reported modest changes in biomarkers of aging. In CALERIE, long-term caloric restriction slowed some DNA-methylation measures of aging, whereas effects on telomere dynamics were limited [117,118]. In a post hoc analysis of DO-HEALTH, omega-3 supplementation modestly slowed several DNA methylation measures of biological aging, whereas additive effects of omega-3, vitamin D, and exercise were observed for PhenoAge [181]. A short exercise intervention also modestly altered a proteomic aging score in men [182]. Multiomic studies of exercise-trained older adults have found fewer age-associated molecular differences in skeletal muscle, better preservation of energy-metabolism programs, and stronger responses to acute exercise. However, observational comparisons are susceptible to selection effects and lifelong behavioral confounding [183]. Collectively, these findings suggest that lifestyle can modify some aging-related biomarkers, but they do not demonstrate broad modification of human aging or establish biological-age clocks as surrogate measures of clinical benefit.

Exercise has strong evidence for improving physical function and reducing the risk of major chronic diseases. Its type, intensity, and duration should be adapted to fitness, frailty, disability, and comorbidity [184]. These benefits may involve improved resolution capacity, but they do not by themselves validate the failed-resolution framework. The term “geroprotector” refers to an intervention proposed to preserve resilience or delay age-related dysfunction. Gerotherapeutics are interventions intended to modify specific mechanisms of aging or treat conditions driven by those mechanisms. These categories overlap, and neither designation establishes efficacy, safety, or regulatory approval. Personalized geroscience should therefore begin with established measures, including physical activity, adequate nutrition, sleep, vaccination, smoking cessation, and appropriate treatment of hypertension, dyslipidemia, and diabetes. Candidate geroprotectors and experimental gerotherapeutics should complement, rather than replace, proven prevention and medical care. Their broader use requires evidence of clinically meaningful benefit and acceptable long-term safety from well-designed trials.

## 6. Clinical Translation and Trial Design

Geroscience proposes that modifying shared aging biology can delay several diseases and functional losses. The clinical attraction is clear because older adults develop interacting multimorbidity rather than isolated disorders. The methodological burden is equally substantial. Trials require acceptable preventive safety, feasible duration, clinically meaningful endpoints, and biomarkers that respond before irreversible disability. Composite endpoints can increase efficiency, but every component must be clinically relevant, consistently defined and ascertained, and analyzed transparently [14,185]. The failed-resolution model adds a specific requirement: treatment should restore the ordered transition from damage response to clearance and functional recovery rather than merely reduce one static marker. A failed-resolution trial should enroll participants with evidence that the targeted defect is present. A senolytic trial should prespecify context-appropriate measures of senescent-cell burden and target engagement, using validated assays where available [31,51]. A STING inhibitor trial should document sterile pathway activation and exclude uncontrolled infection. An efferocytosis intervention should include a functional clearance assay. A mitochondrial intervention should demonstrate impaired respiratory reserve, substrate switching, or mitophagy-related dysfunction. Enrichment can improve power and reduce unnecessary exposure, but thresholds must be validated prospectively and should not be chosen after outcomes are known.

Outcomes should span mechanism, recovery dynamics, function, clinical events, and safety. Mechanistic endpoints may include cell-free nucleic acids, SASP and interferon signatures, inflammasome products, lipid mediators, immune-cell states, matrix turnover, and organ-specific age measures. Dynamic endpoints should quantify recovery after a standardized challenge. Functional endpoints may include gait speed, strength, cardiorespiratory fitness, cognition, vaccine response, wound recovery, and patient-reported function. Clinical endpoints should include incident disease, multimorbidity accumulation, hospitalization, disability, and mortality. Randomization, allocation concealment, blinded outcome assessment, prespecified estimands, adequate power, multiplicity control, and intention-to-treat analysis remain indispensable. Mechanistic mediation should be tested, not inferred from parallel biomarker and functional changes.

Safety is a primary constraint on interventions intended to modify aging in humans. Chronic or systemic inhibition of cGAS-STING could compromise antiviral defense and tumor immune surveillance, NLRP3 inhibition could impair antimicrobial and injury responses, and sustained mTOR suppression could adversely affect immune competence, metabolism, wound healing, or regeneration. These risks will depend on pathway selectivity, dose, treatment schedule, tissue exposure, age, comorbidity, and baseline immune function. Senolytic interventions present a major challenge. Senolytics induce the death of susceptible senescent cells, but they do not themselves remove the resulting cellular material. Benefit may therefore depend on both selective target-cell elimination and sufficient efferocytic capacity. Inadequate clearance could replace a burden of viable senescent cells with apoptotic debris and secondary inflammatory signaling. Indiscriminate elimination of p16-high cells, including liver sinusoidal endothelial cells, caused fibrosis and perivascular damage in mice, indicating that some p16-high populations are functionally required [186]. Future senolytic strategies should consequently evaluate cell-type specificity, tissue distribution, clearance kinetics, and functional recovery rather than senescent-cell markers alone.

The possibility of debris-associated inflammation after senolysis is a mechanistic safety hypothesis, not an established adverse outcome in human trials [7,130,131,132]. A tissue-accessible substudy could assess senescence markers, target-cell death, retained apoptotic material, phagocyte function, inflammatory signaling, and tissue function at baseline, during the expected drug-specific death response, and after recovery. Timing should follow pharmacokinetics and pilot data rather than assume a universal 48–72 h peak. Blinded three-dimensional imaging should distinguish extracellular apoptotic material from material internalized by identified phagocytes, using morphology and complementary death markers. Report extracellular apoptotic-cell density per unit tissue area or volume and the proportion of evaluable apoptotic cells located within phagocytes, using all evaluable apoptotic cells as the denominator. Report phagocyte density separately. TUNEL positivity or proximity to a macrophage alone does not demonstrate failed or successful efferocytosis. Paired ex vivo uptake assays can complement imaging, but neither a static corpse count nor an uptake assay establishes in vivo clearance flux. Serial cell-free DNA and cytokines may support safety monitoring, although they are nonspecific and cannot distinguish increased cell death from decreased clearance by themselves [7]. Clinical adverse events and organ-function deterioration should guide safety decisions. No validated cutoff currently permits a debris index or interferon signature to serve as an automatic stopping rule.

Cell-based products can cause immune reactions, microvascular obstruction, thrombosis, ectopic tissue formation, or tumorigenicity. Epigenetic resetting may disrupt cell identity, promote dysplasia, or increase cancer risk if reprogramming is excessive, prolonged, or incompletely controlled. Safety monitoring should be tailored to the intervention but may include infection, malignancy, wound healing, hematologic indices, liver and kidney function, metabolism, and, when mechanistically justified, clonal hematopoiesis. Preventive interventions in comparatively healthy adults require a substantially higher safety threshold than treatments for severe age-related disease. Prolonged follow-up is essential when an intervention could alter oncogenesis, clonal selection, or tissue identity. Regulatory evaluation will require analytically valid biomarkers, reproducible manufacturing, and interpretable benefit-risk relationships. Biomarkers should not be treated as surrogate outcomes unless intervention-induced changes reliably predict clinically meaningful benefit. Current recommendations emphasize analytical validation, biological and clinical validity, reproducibility across populations, and evidence that biomarker responses are relevant to health or function [115,116]. Population-level translation will additionally require affordability, scalability, and equitable access [187]. A statistically significant change in a molecular aging score has limited public-health value if it is not accompanied by validated evidence of improved function, reduced morbidity, or prolonged healthspan.

## 7. Conclusions

The wound analogy motivates a testable model in which impaired clearance, mistimed damage responses, and incomplete reconstruction contribute to persistent tissue dysfunction. With advancing age, responses that initially support defense and repair may persist or become poorly coordinated, limiting recovery and increasing vulnerability to subsequent injury. The framework complements the hallmarks of aging and evolutionary theories without reducing human aging to a wound. Evidence supporting specific mechanisms is predominantly preclinical, and their contribution to human aging requires further evaluation. Prospective studies can determine whether resolution competence and recovery dynamics improve prediction beyond resting biomarkers and biological-age estimates. Intervention studies can test whether correcting defined defects produces durable functional benefits without compromising host defense, tumor suppression, or acute repair. Lower inflammatory markers or younger molecular-age scores alone do not demonstrate restored tissue function. The framework’s value ultimately depends on whether it explains persistent dysfunction and identifies effective ways to preserve tissue function and reduce age-associated disability.

## Figures and Tables

**Table 1:** Major transitions from successful tissue repair to age-associated failed resolution.

Transition	Successful Repair	Failed Resolution	Testable Readout
Damage signaling	Local and transient; declines after the insult is controlled	Recurrent or persistent DAMP and stress signaling	Signal amplitude and decay after challenge
Inflammatory response	Proportionate recruitment followed by active termination	Persistent, mistimed, or spatially inappropriate activation	Immune-cell kinetics; inflammatory-resolution mediators
Clearance	Efficient intracellular disposal, efferocytosis, and removal of senescent cells	Residual debris, damaged organelles, retained neutrophils, or senescent cells	Autophagic flux; efferocytosis; clearance half-life
Reconstruction	Vascular repair, matrix normalization, and replacement of lost cells	Fibrosis, microvascular dysfunction, niche distortion, or inadequate cell replacement	Matrix turnover; perfusion; regenerative reserve
Functional outcome	Return toward tissue-specific structure and function	Persistent functional deficit and greater vulnerability to subsequent stress	Recovery completeness; organ function; response to repeated challenge

**Table 3:** Failure modes and candidate intervention strategies within the failed-resolution framework.

Dominant Failure Mode	Therapeutic Aim	Representative Approaches	Evidence and Caution
Excess or recurrent damage input	Reduce preventable injury and preserve physiological reserve	Risk-factor control, vaccination, exercise, adequate nutrition, and exposure reduction	Strongest human evidence for health benefit; does not by itself validate the framework
Persistent danger sensing	Reduce maladaptive signaling while preserving acute defense	Biomarker-selected cGAS–STING, NLRP3, or JAK–STAT modulation	Mainly preclinical; infection, repair, and immune-surveillance risks
Senescent-cell accumulation	Remove harmful cells or restrain their secretory phenotype	Senolytics, senomorphics, immune-mediated targeting	Early human feasibility; heterogeneous targets and on-target tissue risks
Clearance failure	Restore efferocytosis and removal of cellular remnants	Macrophage metabolic support, pro-resolving mediators, clearance-pathway modulation	Strong mechanistic rationale; limited human validation and risk of inappropriate phagocytosis
Quality-control and metabolic failure	Restore context-dependent organelle turnover and metabolic flexibility	mTOR/AMPK modulation, NAD+-directed treatment, autophagy or mitophagy enhancement	Target engagement is common, but durable functional benefit has not been established
Regenerative or niche failure	Rebuild tissue without loss of identity or tumor control	Matrix and vascular repair, cell-based therapy, controlled epigenetic resetting	Predominantly experimental; manufacturing, ectopic-tissue, and oncogenic risks

## Data Availability

No new data were created or analyzed in this study. Data sharing is not applicable to this article.

## Abbreviations

AMPK	AMP-activated protein kinase
ATP	adenosine triphosphate
BAZ2B	bromodomain adjacent to zinc finger domain 2B
BRD4	bromodomain-containing protein 4
CAR	chimeric antigen receptor
cGAMP	cyclic guanosine monophosphate–adenosine monophosphate
cGAS	cyclic GMP–AMP synthase
COPD	chronic obstructive pulmonary disease
CRP	C-reactive protein
DAMP	damage-associated molecular pattern
ECM	extracellular matrix
EP2	prostaglandin E2 receptor 2
HSP90	heat-shock protein 90
IL	interleukin
JAK–STAT	Janus kinase–signal transducer and activator of transcription
LINE-1	long interspersed nuclear element 1
MAPK	mitogen-activated protein kinase
MRE11	meiotic recombination 11 homolog 1
mtDNA	mitochondrial DNA
mTOR	mechanistic target of rapamycin
NAD+	nicotinamide adenine dinucleotide
NF-κB	nuclear factor kappa B
NLRP3	NLR family pyrin domain containing 3
NMN	nicotinamide mononucleotide
NR	nicotinamide riboside
OSK	OCT4–SOX2–KLF4
PGE2	prostaglandin E2
QPCTL	glutaminyl-peptide cyclotransferase-like protein
ROS	reactive oxygen species
SASP	senescence-associated secretory phenotype
SIRT	sirtuin
STING	stimulator of interferon genes
TBK1–IRF3	TANK-binding kinase 1–interferon regulatory factor 3
TGF-β	transforming growth factor beta
TNF	tumor necrosis factor
TREX1	three-prime repair exonuclease 1
uPAR	urokinase-type plasminogen activator receptor
YAP/TAZ	Yes-associated protein/transcriptional coactivator with PDZ-binding motif
